# Tunable electronic properties of graphene through controlling bonding configurations of doped nitrogen atoms

**DOI:** 10.1038/srep28330

**Published:** 2016-06-21

**Authors:** Jia Zhang, Chao Zhao, Na Liu, Huanxi Zhang, Jingjing Liu, Yong Qing Fu, Bin Guo, Zhenlong Wang, Shengbin Lei, PingAn Hu

**Affiliations:** 1 Key Laboratory of Micro-systems and Micro-structures Manufacturing, Ministry of Education, Harbin Institute of Technology, No. 2 Yikuang Street, Harbin, 150080, China; 2 Faculty of Engineering & Environment, Northumbria University, Newcastle upon Tyne, NE1 8ST, UK; 3 Institute of Fundamental and Frontier Sciences, University of Electronic Science and Technology of China, No. 4, North Jianshe Road, Chengdu, China; 4 State Key Laboratory of Robotics and System, Harbin Institute of Technology, No. 2 Yikuang Street, Harbin, 150080, China

## Abstract

Single–layer and mono–component doped graphene is a crucial platform for a better understanding of the relationship between its intrinsic electronic properties and atomic bonding configurations. Large–scale doped graphene films dominated with graphitic nitrogen (GG) or pyrrolic nitrogen (PG) were synthesized on Cu foils via a free radical reaction at growth temperatures of 230–300 °C and 400–600 °C, respectively. The bonding configurations of N atoms in the graphene lattices were controlled through reaction temperature, and characterized using Raman spectroscopy, X–ray photoelectron spectroscopy and scanning tunneling microscope. The GG exhibited a strong n–type doping behavior, whereas the PG showed a weak n–type doping behavior. Electron mobilities of the GG and PG were in the range of 80.1–340 cm^2^ V^−1^·s^−1^ and 59.3–160.6 cm^2^ V^−1^·s^−1^, respectively. The enhanced doping effect caused by graphitic nitrogen in the GG produced an asymmetry electron–hole transport characteristic, indicating that the long–range scattering (ionized impurities) plays an important role in determining the carrier transport behavior. Analysis of temperature dependent conductance showed that the carrier transport mechanism in the GG was thermal excitation, whereas that in the PG, was a combination of thermal excitation and variable range hopping.

Graphene, a two–dimensional crystal with carbon (C) atoms packed in a hexagonal lattice, has attracted extensive research attention in the past decade, owing to its extraordinary properties^1^,^2^,^3^,^4^. The deliberate incorporation of heteroatoms into the graphene lattice could offer an efficient way to tailor its electronic^5^, chemical^6^, optical^7^ and magnetic^8^ properties, and therefore broaden their applications. As one of most promising dopants, nitrogen (N) could be easily incorporated into the graphene, thus creating charged sites and resulting in redistribution of the spin and charge density states in the graphene^8^,^9^,^10^,^11^,^12^,^13^. Commonly, three bonding configurations appear in the N–doped graphene lattice: (1) one C atom is substituted with one graphitic N, which is then bonded to other three adjacent C atoms in the hexagonal ring; (2) pyridinic N is bonded with two C atoms at the edges or defects of graphene, which contributes one p electron to the π system; (3) pyrrolic N identifies to N atoms that contributes two p electrons to the π system^14^. Obviously, the bonding configuration of the N atoms in the graphene lattice will significantly influence its physical and chemical properties, such as electron, hole and phonon transport behaviors. In the graphitic configuration, the excess valence electrons are delocalized, thus leading to an n–type doping effect^15^,^16^, whereas either pyridinic or pyrrolic N atoms show a p–type doping effect^16^,^17^,^18^. Due to the charges, impurities, scattering and screening effects, novel transport behaviors of carriers can be obtained, such as the wide scale linear dependence of conductivity on the applied gate voltage^19^,^20^ and the mobility asymmetry between electrons and holes^19^. Also, the carrier density inhomogeneity induced by the charged impurities will shift the position of minimum conductivity point^21^. However, previous studies were unlikely to rule out the influences of stacking patterns, electronic structures and couplings since the multilayer graphene combining with multi–components nitrogen was adopted^5^. Therefore, synthesis of a single bonding configuration of N doped monolayer graphene is of vital importance to reveal the influence of N bonding configuration on redistribution of the spin and charge states, as well as the electronic structures of the doped graphene. Meanwhile, the single bonding configuration for the N doped graphene film is expected to be an excellent platform for electronic devices, oxygen reduction reaction, batteries, sensors and supercapacitors^9^,^10^,^11^,^12^,^13^.

Chemical vapor deposition (CVD) is a powerful method among the synthesis techniques of heteroatom doped graphene films^9^,^10^,^14^. During the synthesis process, the bonding configurations for the N atoms are strongly influenced by the growth parameters, such as precursors, catalysts, flow rates and growth temperatures. By using CH_4_/NH_3_ as precursors, graphitic N dominated graphene was synthesized on Cu foils at 800–1000 °C^9^,^15^, whereas pyridinic N or pyrrolic N dominated graphene was grown on Ni foils^22^. Impressively, pure pyridinic N doped graphene was grown on Cu foils using C_2_H_4_/NH_3_ as the precursors^23^,^24^. However, few research has been focused on synthesis of single bonding configuration for N doped graphene, and the effects of N bonding configurations on the electronic properties of the doped graphene are not well understood.

Herein, we proposed a temperature controlled method for N–doped graphene film growth on Cu foils via a free radical reaction. The graphene was predominantly doped with graphitic and pyrrolic N atoms at different temperatures of 230–300 °C and 400–600 °C, respectively. The bonding configurations of N atoms in the graphene lattice were characterized using Raman spectroscopy, X–ray photoelectron spectroscopy (XPS) and scanning tunneling microscope (STM). The graphitic N doped graphene exhibited a strong n–type transport behavior, whereas the pyrrolic N doped graphene showed a weak n–type transport behavior with a symmetric electron–hole transport. Analysis of temperature dependent conductance showed that the carrier transport mechanism was a thermal excitation process for the graphitic N dominated graphene, whereas it was a combination of thermal excitation and variable range hopping for the pyrrolic N dominated graphene. As–grown mono–component doped graphene film is an important platform for the understanding of the relationship between the bonding configurations and its intrinsic physics of N–doped graphene, as well as development of graphene–based devices.

## Results and Discussion

### Growth of N–doped graphene

**Figure 1:**
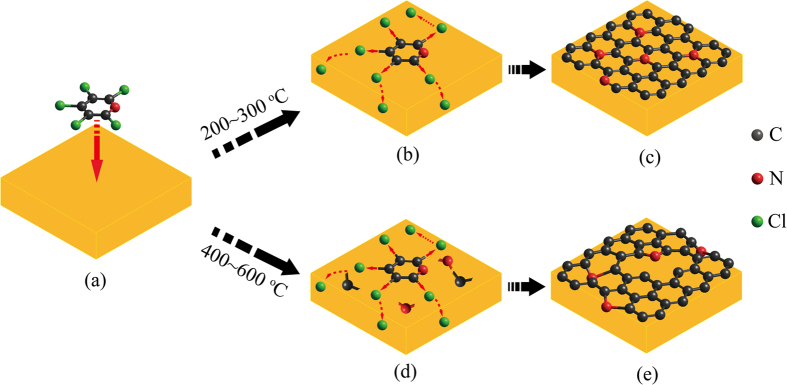
Schematic illustration of growth of graphitic N dominated graphene (GG) at 200–300 °C (**a–c**) and pyrrolic N dominated graphene (PG) at 400–600 °C (**a**,**d**,**e**), respectively, via the free radical reaction.

Growth of the N–doped graphene film on Cu foils via the free radical reaction was previously reported by our group^25^. In brief, the pentachloropyridine molecules were firstly adsorbed on the Cu foils (Fig. 1a); then the coupling reaction occurred between halides and copper to form aryl–aryl and carbon–nitrogen bonds (Fig. 1b,d), which favored a bottom–up formation of network carbon structures (Fig. 1c,e). The dechlorination was bonded to copper, thus forming copper chloride compounds (Fig. 1b,d), which was confirmed using high–resolution XPS scan of the Cl2p peak^25^. Upon the free radical reaction, the graphene shows a higher growth rate compared with that grown at a higher temperature (≥800 °C)^25^. A continuous N–doped graphene film covered on the whole Cu foil was achieved within 5 min using this method.

**Figure 2:**
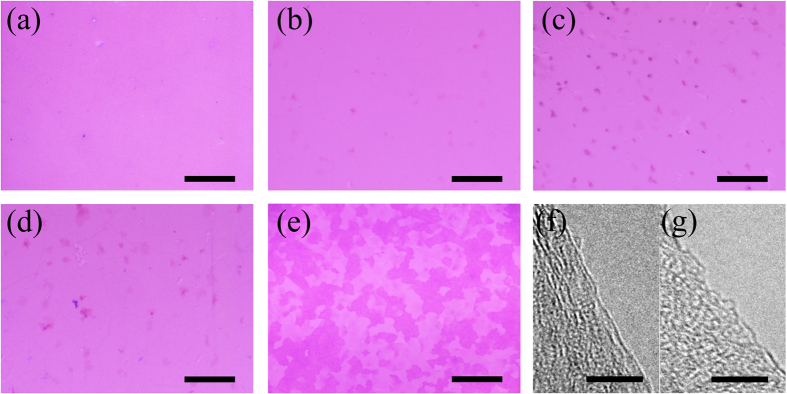
(**a–e**) Optical images of transferred N–doped graphene on the 300 nm SiO_2_/Si substrate growth at temperature in the range of 230–600 °C, scale bar = 20 μm. (**f**,**g**) High–resolution TEM image of single layer graphene grown at 300 °C and 500 °C, scale bar = 5 nm.

**Figure 3:**
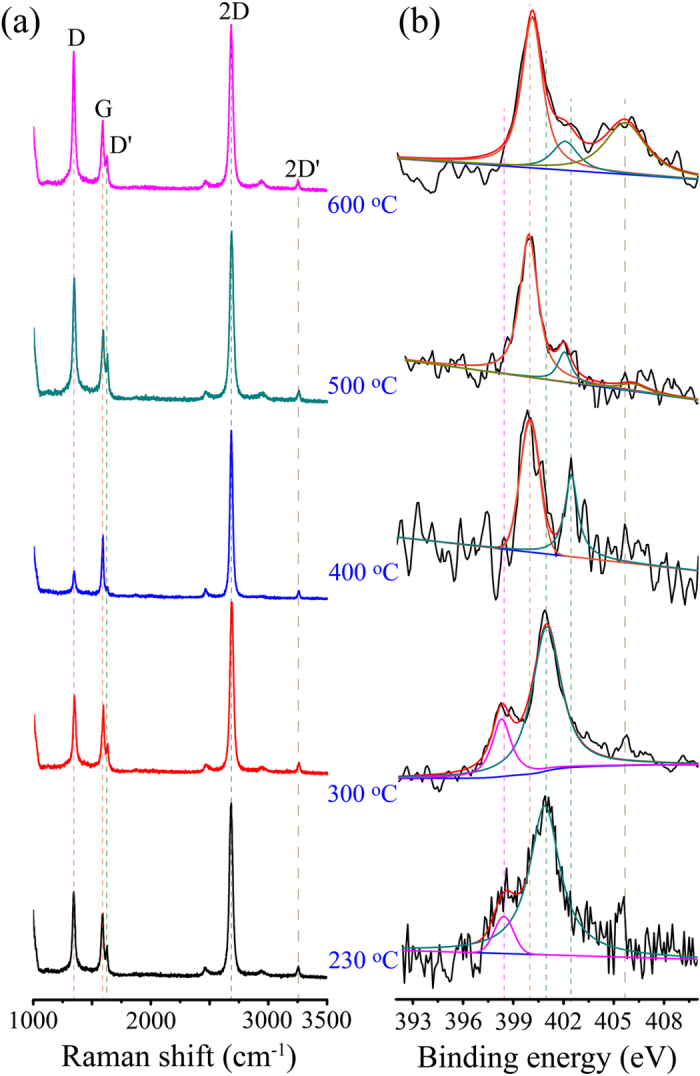
(**a**) Raman spectra of N–doped graphene grown at temperatures in the range of 230–600 °C. (**b**) High–resolution XPS scan of N1s of doped graphene grown at different temperatures (230–600 °C).

To reveal the influence of temperature on the growth of N–doped graphene via the free radical reaction, the Cu foil substrates were kept at various temperatures between 230 °C and 600 °C for growth. The morphology of the resultant film was characterized using SEM (Fig. S1) and optical microscopy (Fig. 2a–e). Clearly, a continuous graphene film can be obtained on the Cu foils at each temperature. Some additional graphene layers appear on the top of this graphene film, which can be inferred from the different contrasts in the optical images (Fig. 2b–e). The free radical reaction will be speeded up at an elevated temperature, resulting in formation of a large quantity of radicals and formation of multilayer N–doped graphene grains simultaneously. It should be noted that a poor quality film was obtained on the Cu foils when the growth temperature was up to 600 °C. We assume that the vigorous reaction between chlorine free radicals and copper makes a rough copper surface and hinders the formation of a high quality graphene film. AFM measurements were performed to determine the layer feature of the N–doped graphene films (as shown in Fig. S2). The thicknesses of the doped graphene films were in the range of 0.8–1.6 nm, which indicated a single–layer feature considering the deviation in the AFM measurements^1^,^26^,^27^. Furthermore, high–resolution TEM image at the back–folded of edge from each doped graphene clearly shows a single–layer feature. Selected high–resolution TEM images of the doped graphene grown at 300 °C and 500 °C are shown in Fig. 3f,g, respectively. The single–layer feature is critical for the electronic properties, which will be discussed in the later section.

### Characterization of the bonding configurations of nitrogen atoms in graphene

To further investigate the effect of growth temperature on the vibrational modes of the graphene lattice, we performed micro–Raman spectroscopy on the samples grown at different temperatures (Fig. 3a). The peaks at ~1590 cm^−1^ and ~2680 cm^−1^ are assigned to the G band and 2D band, respectively, which confirm the presence of graphitic carbon^28^. The peak at ~1340 cm^−1^ activated by the defects via an intervalley double–resonance process is assigned to D band^28^. Generally, the intensity ratio of D band over G band (I_D_/I_G_) reveals the degree of defects and in–plane crystallite size (L_D_). Herein, the ratio (I_D_/I_G_) decreases as the temperature is increased in the range of 230–400 °C (Fig. 3a, Table S1), but then increases as the temperature is above 400 °C (Fig. 3a, Table S1). As a result, a relatively better quality of N–doped graphene was obtained at 400 °C via the free radical reaction. Alternatively, the largest L_D_ value further highlights the good quality of the N–doped graphene grown at 400 °C (Table S1). One hypothesis is that the pyridinic and chlorine free radicals might form after the breaking of carbon–chlorine bonds upon the coupling reaction^29^,^30^. The occupation of chlorine adatoms on the flat terraces of copper will prevent the absorption and decomposition of the precursors. Therefore, the competitive adsorption between the chlorine adatoms and precursors will significantly affect the growth process and quality of doped graphene. Increasing the substrate (Cu foils) temperature and using a lower pressure during reaction will remove the chlorine adatoms effectively, which facilitates the growth of doped graphene^29^,^30^. In this scenario, the quality of N–doped graphene is improved at the elevated temperature (≤400 °C), but becomes deteriorated at a much higher temperature (500–600 °C) owing to the strong etching on the copper surface. In addition, a lower pressure (~15 torr) was employed to remove the chlorine adatoms efficiently and was favored for growing high quality N–doped graphene films.

It is noteworthy that an obvious peak at ~1625 cm^−1^ accompanied with a peak at ~3250 cm^−1^ appears in each N–doped graphene sample (Fig. 3a), which is absent in the pristine graphene^28^. These peaks are assigned to D′ and 2D′ bands, which arise from the intravalley, defect–induced double–resonance process^15^,^31^. The heteroatoms (i.e. N atoms) incorporated in graphene lattice will induce the D′ and 2D′ bands^10^,^12^,^15^,^25^ as demonstrated in Fig. 3a.

The position and shape of Raman peaks can also provide doping information in the graphene. Herein, the position of G band was upshifted to the range of 1588.1–1589.0 cm^−1^ for the GG (Table S1) compared with that at 1580 cm^−1^ for the pristine graphene^32^,^33^. Meanwhile, the G band became softening with full width at half maximum (fwhm(G)) in the range of 20.3–29.2 cm^−1^ compared with that of 6–16 cm^−1^ in the pristine graphene^32^. Both these changes confirmed an n–type doping feature in the GG samples^32^,^33^. For the position of 2D band, there was about 17.2–20 cm^−1^ downshift compared with that in the pristine graphene (2700 cm^−1^). The downshift of position agrees well with the electron doping feature in the graphene. Otherwise, the change of the fwhm(2D) appeared to be irregular with a larger bandwidth, which can’t be applied alone to identify the doping effect^14^.

To determine the bonding configuration of incorporated N atoms in the graphene, XPS analysis was performed on each sample. In the C1s spectra (Fig. S3), the peak at 284.5 ± 0.1 eV represents the *sp*^2^ hybridized C–C bond (C1). The peaks at 285.4 ± 0.2 eV and 286.4 ± 0.1 eV correspond to the trigonal phase with a *sp*^2^ C (C2) and the tetrahedral phase with a *sp*^3^ C (C3), respectively^9^,^22^,^23^. The other peak at 288.5 ± 0.5 eV is identified as the CO– type bonds (C4)^24^. At lower growth temperatures of 230–300 °C, the C1s spectra can be well fitted using three Gaussian curves. Apart from the C1 and C4 curves, only one peak at 285.4 ± 0.2 eV is fitted, which refers to N–*sp*^2^ C bond (C2). It may be come from pyridinic N or graphitic N atoms in graphene framework as well as the nitro– group connected to graphene framework, considering the spatial and electronic structure after incorporated these N atoms^16^,^26^. These bonding configurations could be identified precisely in compared with N1s spectrum further^26^. But for the higher growth temperatures of 400–600 °C, an addition peak centered at 286.4 ± 0.1 eV can be fitted, which represents the N−*sp*^3^ C bond (C3). This bonding is originated from new type of incorporation N atoms other than pyridinic N or graphitic N atoms. Meanwhile, this bonding will increase the structural distortion by disrupting the plane framework of graphene. It would be assigned to pyrrolic N bonding configuration. The relative content of each component (Table S2) clearly shows the rates of different bonding.

As an alternative, the different bonding configurations of N atoms at different growth temperatures are shown in Fig. 3b and Table 1. At a low temperature range (e. g., 230–300 °C), the peak at 400.9 eV and ~398.3 eV could be assigned to graphitic N and pyridinic N^14^,^25^,^34^,^35^, respectively, considering the only N–*sp*^2^ C bonding configuration confirmed in the C1s spectra (Fig. S3). In this condition, the incorporated N atoms have the *sp*^2^ hybridization with two or three adjacent carbon atoms and vacancies^14^,^25^,^34^,^35^. Furthermore, the dominant graphitic N can be revealed from the ratio of the two components (Table 1). At higher temperatures (e. g., 400–600 °C), The main peak at ~399.9 eV is assigned to pyrrolic N^9^,^14^, while the other peak in the range of 401.8–402.4 eV is assigned to graphitic N^13^,^14^. The assignments can be understood via the structural and electronic distort induced by disrupting the plane framework of graphene accompanying with the formation of N−*sp*^3^ C bond. Apart from these three types, an obvious peak at 405.6 ~ 406.1 eV is assigned to oxidation of N groups in the graphene^36^, which was obtained at 500 ~ 600 °C, but was negligible for the other samples grown at 230–400 °C. The same trend of the peak location and nitrogen content has been found in the other samples (Fig. S4 and Tables S3).

These bonding configurations of N atoms are determined by bonding energies and their thermal stabilities. The coupling reaction could reduce the energy barrier for dechlorination and leave pyridinic ring radicals intact at low reaction temperatures (e. g. 230 °C, 300 °C), although the bonding energy of C–Cl (339 kJ mol^−1^) is higher than that of C–N (305 kJ mol^−1^)^37^. The existing pyridinic radicals have been proved by the electron paramagnetic resonance test^25^. The highly reactive radicals of pyridinic ring were easily bonded each other to form graphitic N dominant graphene with a few of pyridinic N at edges or structural vacancies. XPS scans of N1s spectra at 230 °C and 300 °C have provided an evidence for the process. As the reaction temperature was increased, the breaking pyridinic rings produced more free carbon, nitrogen and alkyl radicals, and subsequently graphene film was formed through a series of reactions among these radicals. In this condition, a lower energy was responsible for the formation of pyrrolic N compared that more activation energy was needed to form pyridinic N and graphitic N. Meanwhile, the weak thermal stability of pyridinic N will be gradual pyrolysis and leave graphitic N residues. Thus, XPS scans of N1s spectra at 400–600 °C show a predominant pyrrolic N with a small part of graphitic N.

It is noted that there is minor shift of peak position of graphitic N as the growth temperature increasing. This shift can be attributed to the different environments of nitrogen atoms created by the dopants, defects, vacancies and uniformity in doped graphene^38^. Actually, a larger peak position variation of graphitic N in the range of 398.3–401.9 eV has been observed in the carbon–nitrogen compounds^39^. In addition, these changes could be also attributed to conditions of the substrate and thermal stability of each bonding configuration of nitrogen^40^,^41^. Previous reports proved the decisive role of substrate for hemolysis of covalent carbon–halogen bonds at an elevated temperature and subsequent association of radicals^41^. The activation of substrate was important for the confinement of molecular motions in two dimensions. The pre–homolysis of C–Cl bonds leaves pyridinic rings intact and carbon fragments which form the carbon network at relative lower temperatures of 230 °C and 300 °C^40^. Simultaneously, the chloric radicals react with copper and form compounds, thus reducing the activation of catalysts. The lowest energy geometry of hexagon carbon network would be broken down at a higher temperature considering defects such as polygons, distortion and high stress in rings. It’s not invertible for the formation of copper–chloride compounds^41^.

Breaking pyridinic ring at elevated temperatures would generate more carbon and nitrogen fragments. Less N atoms could be incorporated into graphene lattice via C–N bond at relatively high temperature because of its low thermal stability. As a result, the content of nitrogen was decreased as temperature was increased in the range of 230–500 °C, which was well consistent with Liu’s findings^42^. Higher reaction temperature(≥500 °C) resulted in formation of rougher terraces on copper by the strong etching (Fig. 2e and Fig. S1e). The rough surface could induce rugged N–doped graphene film that easily absorbed more pentachloropyridine molecule, leading to higher nitrogen content in graphene (shown in XPS characterization, e.g. 8.2 at % at 600 °C).

As a result, the content of N configurations was normalized by the graphitic N and pyrrolic N and the results are summarized in Table 1. The analysis of XPS confirmed that the graphitic and pyrrolic N–doped graphene have been successfully synthesized at 230–300 °C and 400–600 °C, respectively. Hereafter, the abbreviations of GG and PG are used for each type of doped graphene.

**Figure 4:**
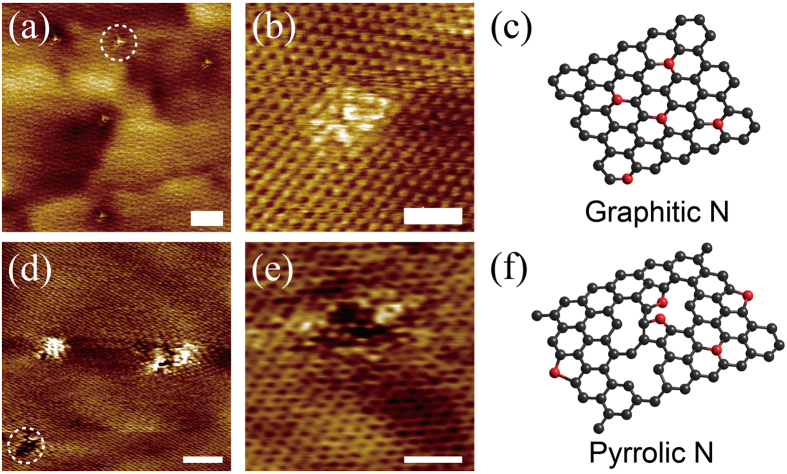
STM images of N–doped graphene dominant with graphitic N and pyrrolic N. (**a**) Large–scale STM topographic image of graphitic N dominated graphene on Cu foil showing the presence of pointlike N dopants (V_bias_ = −0.015 V, I_set_ = 0.3 nA). Scale bar = 5 nm. (**b**) High–resolution STM image of graphitic N dopant (V_bias_ = −0.015 V, I_set_ = 0.3 nA). Scale bar = 1 nm. (**c**) Scheme of graphitic N. (**d**) Large–scale STM topographic image of pyrrolic N dominated graphene on Cu foil (V_bias_ = −0.02 V, I_set_ = 0.3 nA). Scale bar = 2 nm. (**e**) High–resolution STM image of pyrrolic N dopant (V_bias_ = −0.02 V, I_set_ = 0.3 nA). Scale bar = 1 nm. (**f**) Scheme of pyrrolic N. The red and black balls in (**c**,**f**) represent nitrogen and carbon atoms, respectively.

The bonding configurations of N atoms in graphene lattice were directly imaged using STM measurements. A large–area STM image (Fig. 4a) of the GG (growth at 300 °C) on the Cu foil reveals the clear honeycomb lattice decorated by several triangular bright spots. High–resolution STM image (Fig. 4b) provides a close–up topography of an individual N dopant site. The triangular bright spots surrounded a dark site in graphene plane are quite similar to previously reported results^15^,^43^, and is assigned to graphitic N configuration (Fig. 4c). The topography observation suggests a positive charged N in the forming of graphitic N, which was also reported by Yu *et al*.^44^ and Mayer *et al*.^45^. The positive charge can be explained by electron transfer from the N atom to the π conjugated state. If N is positively charged, the surrounding carbon atoms should be negatively charged because of the screening effect. Also, the negatively charged carbon can explain a shift of the localized π state of carbon from the Fermi level to the upper energy level. In contrast, the STM image (Fig. 4d) collected from the PG grown at 500 °C shows a different configuration with a large quantity of structural defects comparing with that shown in Fig. 4a. This can also be confirmed by the high intensity of the D peak in the Raman spectrum (Fig. 3a). High–resolution STM image shown in Fig. 4e exhibits an apparent vacancy defect in the graphene plane, combining with light spots at the edge of vacancy. These light spots in the high–resolution STM image are attributed to the incorporated nitrogen atoms, which are positively charged. A probable scheme of the PG is given in Fig. 4f 43.

### Electronic properties of N-doped graphene

The electrical properties, especially the carrier transport behavior in graphene, are highly relevant to the scattering mechanism at lattice defects, ripples and ionized impurities^5^,^16^. Accordingly, the lattice defects have been demonstrated to be strongly relevant to bonding configurations^16^. Thus, electrical measurements were conducted in both the GG and PG to reveal the role of bonding configurations in the electrical properties of N–doped graphene. The N–doped graphene film was firstly transferred onto silicon oxide substrate and patterned into micoribbons (Fig. S5a), and then bottom–gate FETs were fabricated (Fig. S5b). Since both types of the doped graphene are mainly single layer (Fig. S2 and Fig. 2f,g), the influences of stacking patterns, electronic structures and couplings in multilayer graphene sample could be reasonably ruled out^5^. Therefore, the GG and PG are proved to be an ideal platform to precisely investigate the electrical properties of N–doped graphene.

**Figure 5:**
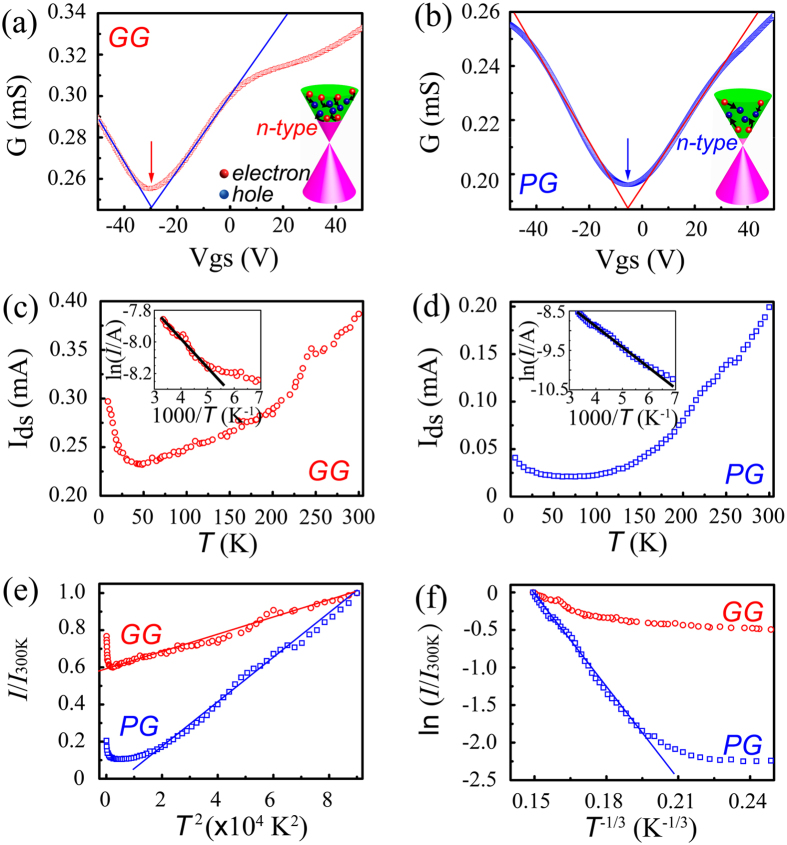
Electrical properties of graphitic N and pyrrolic N dominated graphene. (**a**) Conductance (G) as a function of gate voltage (V_gs_) for GG with G_min_ at −30 V, V_ds_ = 1 V. Inset is energy band of the GG, the red and blue spheres represent the electron and hole, respectively. (**b**) Conductance (G) as a function of gate voltage (V_gs_) for PG with G_min_ at −5.2 V, V_ds_ = 1 V. Inset is energy band of the PG. Drain–source current (I_ds_) with V_ds_ = 1 V and floating gate (V_gs_ = 0 V) of GG (**c**) and PG (**d**), respectively. The inset shows the corresponding Arrhenius plot and yields activation energy. (**e**) Normalized I_ds_ by that at 300 K (I_ds_/I_300K_) as a function of T^2^. (**f**) The I_ds_/I_300K_ as a function of T^−1/3^ with logarithmic coordinates.

The conductance of the doped graphene as a function of applied gate voltage is plotted in Fig. 5a for the GG. Significantly, for V_g_ values near to its minimum, V_g,min_, where the minimum conductance (G_min_) occurs, the conductance is linearly depended on V_g_ in both electron– and hole– transport regimes, indicating a long–range scattering (incorporated impurities) in the GG^19^,^46^,^47^. The asymmetric curves accompany with neutrality points shift negatively towards the gate voltage region of −30–−24.5 V, indicating a strong n–type doping feature. Inset image in Fig. 5a illustrates the energy band of the GG. The incorporated one graphitic N atom will contribute near half of an electron^16^, which boosts the electron concentration and shifts Fermi level to the conduction band (Fig. 5a inset). The hole and electron mobilities were calculated according to the equation: *μ* = (Δ*I*_ds_·*L*/*W*)/(Δ*V*_gs_·*V*_ds_·*C*_ox_), where, *C*_ox_ is the silica gate capacitance (1.15 × 10^−8^ F·cm^−2^ for a gate oxide thickness of 300 nm). As a result, the hole and electron mobilities are in the range of 59.5–429.8 cm^2^ V^−1^·s^−1^ and 80.1–340 cm^2^ V^−1^· s^−1^, respectively. The obtained high mobility of the carriers would be attributed to minimized defect formation upon direct substitution (Fig. 4a–c).

Figure 5b plots the conductance versus gate voltage of the FET devices based on the PG. A more symmetrical curve with neutrality point in the range of −9.5–−5.2 V was obtained, which is close to intrinsic bipolar properties of the pristine graphene but showing a weak n–type doping feature. Comparing to the GG, the lower Fermi level and electron concentration are illustrated in inset scheme of Fig. 5b. The observed n–type doping feature in the PG seems to be in conflict with the previous reports, which showed that pyrrolic N in graphene provided p–type doping effects^5^,^16^,^47^,^48^. Nevertheless, the previous seminal theoretical work^47^ combined with experimental work^5^ tried to explain this conflict via connecting the bonding configurations with electronic structures. Since the nitrogen atom has five valence electrons, three of them form three σ bonds with adjacent two carbon atoms and one hydrogen atom, while the other two electrons form one π bonds in the pyrrolic N. The absence of electron lone pairs forces the additional charge from the N atoms to go to the conduction band, giving rise to n–type doping. This is quite similar to the electronic structure in graphitic N doped graphene (Fig. 4b), which exhibits a strong n–type doping feature^16^. In addition, the remaining graphitic N component in the PG injects extra electrons to the carbon π networks via approximately a half electron per graphitic N atom^16^. Accordingly, the hole and electron mobilities were calculated to be 53.5–134.4 cm^2^ V^−1^·s^−1^ and 59.3–160.6 cm^2^ V^−1^·s^−1^, respectively. The lower carrier mobilities in the PG compared with those in the GG could be attributed to more vacancies and defects introduced by the pyrrolic N, thereby increasing the strong scattering effect.

The crossing of the linear fits (shown in Fig. 5a,b) defines the residual conductance (G_crossing_) at V_g_ = V_g,crossing_ and V_g,crossing_ (indicated by arrow). In our transport measurements, the gate voltage at G_crossing_ is found to be equal to the V_g,min_ (neutrality points), implying the potential fluctuations induced by the randomly distributed charged impurities (herein, N atoms) localize in the graphene system with a homogenously spatial distributions^5^,^49^. This is different from the deviation between the V_g,min_ and V_g,crossing_ observed in some nitrogen and potassium doped graphene^5^. Furthermore, there is a deviation from the linear fitting at high V_g_ regimes for both in the GG and PG (Fig. 5a,b). We believe that the presence of both long–range scaterers (e.g., incorporated nitrogen atoms^42^, ripples) and short–range scatterers (e.g., structural defects, disorders) contribute to the discrepancy of the results.

Carriers transport mechanism can be further investigated by analyzing temperature dependent measurements as shown in Fig. 5c–f. Both types of doped graphenes exhibit insulating behavior with increasing conductance as the temperature increased (Fig. 5c,d). The conductance was decreased with lowering the temperature down to about 50 K, but increased slightly with further lowering the temperature, which are quite similar to those for the single reduced graphene oxide sheet (rGO)^50^ and stacked graphene oxide films^51^,^52^ as well as their composites^53^. At a high temperature (above 200 K), Arrhenius plots shown in Fig. 5c,d yield the activation energy values of 15.4 meV and 44.5 meV for the GG and PG, respectively, indicating a thermal excitation effect exists in both types of doped graphene. The higher activation energy in the PG would be attributed to a larger amount of structural defects and disorders (Fig. 4d,e), consistent with the findings from Raman spectra (Fig. 3a and Table S1) and N1s peak analysis of XPS (Fig. 3b and Table 1, Fig. S4 and Table S3). For the temperature below 50 K, the conductance is metallic–like, similar to that in the rGO^50^,^52^ and exfoliated graphite^54^. In addition, the observed linear relationship between the conductance and quadratic temperature in Fig. 5e also provide an evidence of direct thermal excitation of carriers in both the GG and PG.

Incorporation of the pyrrolic nitrogen into graphene introduced not only defects but also a strong disorder resulting in localization of carriers, which is similar to the carriers in the rGO sheet^50^. These localization phenomena in the PG would determine the carriers transport behavior, showing a slightly decreased conductance at a very low temperature (Fig. 5d), compared with that in the conventional two–dimensional materials^55^. Fig. 5f plots the relationship of the normalized I_ds_/I_300 K_ versus of T^−1/3^ of both types of doped graphene. The linear plot generally shows a signature of variable–range hopping (VRH) transport^50^,^52^. Only the pyrrolic graphene can be linearly fitted (Fig. 5f) suggesting a strong localization °Ccurs. These findings are consistent with the results from the Raman and XPS analysis (Fig. 3a,b). The current change in the PG (Fig. 5d) is increased up to one order as the temperature is increased from 5.2 K to 300 K, which is larger than that in the GG (Fig. 5c), but still smaller than that in the GO sheet and its composite^50^,^53^ due to the degree of defect and disorder.

## Conclusion

We have synthesized graphitic and pyrrolic N dominated graphene films by controlling the growth temperature via the free radical reaction. Raman spectra, XPS and STM results confirm the graphitic N and pyrrolic N dominated graphene films are grown at temperatures in the range of 230–300 °C and 400–600 °C, respectively. Furthermore, the graphitic N dominated graphene exhibits a strong n–type doping effect, whereas the pyrrolic N dominated graphene shows a weak n–type doping effect. The enhanced doping effect in graphitic N dominated graphene results in an asymmetry electron–hole transport which suggests that the scattering by ionized incorporated N impurities plays an important role in determining the carrier transport behavior. Temperature dependent conductance shows carriers transport mechanism in the GG was thermal excitation, whereas that in the PG was a combination of thermal excitation and variable range hopping.

## Methods

### Synthesis of N–doped graphene

Cu foils (Alfa Aesar, 25 μm thick, 99.8% purity) and pentachloropyridine (Aladdin, 98%) were separately placed in a furnace with two–temperature zones. Prior to the growth of graphene, the Cu foils were annealed at 1000 °C for 20 min in H_2_ atmosphere (90 sccm) at 15 torr, followed by cooling down to the designated temperature (230–600 °C) and then the pentachloropyridine was heated to 130 °C and kept for various durations (0.5–5 min). After growth, the furnace was cooled down to room temperature. As–grown N–doped graphene was transferred onto the SiO_2_/Si substrates using a PMMA supported method for the characterization and device fabrications.

### Characterization

As–grown N–doped graphene was characterized using scanning electron microscopy (SEM, Hitachi SU8000, with an accelerating voltage 15 kV), transmission electron microscopy (TEM, Tacnai–G2 F30, accelerating voltage of 300 kV), optical microscopy (Leica DM4500P), Raman spectroscopy (LabRAM XploRA, incident power of ~1 mW, pumping wavelength of 532 nm), atomic force microscopy (AFM, multimode Nanoscope IIIa, tapping mode), X–ray photoelectron spectroscopy (XPS, Thermo Scientific K–Alpha XPS, using Al (Ka) radiation as a probe) and scanning tunneling microscope (STM, Agilent 5100). Raman spectra, optical and AFM images of all the doped graphene were collected from the transferred doped graphene onto the SiO_2_/Si substrates without further annealing. The SEM images, STM images and XPS data were collected directly from all the doped graphene on Cu foils after growth.

### Preparation of field effect transistors and electrical measurements

Electrical properties of the N–doped graphene films were evaluated based on a field effect transistor (FET) configuration. The N–doped graphene film was firstly transferred onto a SiO_2_/Si substrate with a 300 nm thermal oxide layer, and then patterned into micro–ribbons^24^. The bottom–gate FETs were made via thermal evaporation of Cr/Au contacts (10/90 nm) onto the micro–ribbons. The channel length (*L*) and width (*W*) were measured to be 14 μm and 24.5 μm, respectively. To obtain a better contact, the devices were thermally annealed at 200 °C in H_2_/Ar (10/20 sccm) atmosphere for 20 min. Electrical measurements were carried out in a vacuum after pumping for 12 hours at room temperature using a semiconductor analyzer (Keithley 4200–SCS) combined with a Lakeshore probe station. Low temperature measurements were carried out from 5.2 K to 300 K in a vacuum of 1.58 × 10^−6^ torr.

## Additional Information

**How to cite this article**: Zhang, J. *et al*. Tunable electronic properties of graphene through controlling bonding configurations of doped nitrogen atoms. *Sci. Rep.* **6**, 28330; doi: 10.1038/srep28330 (2016).

## Supplementary Material

Supplementary Information

## Figures and Tables

**Table 1:** Detailed parameters of N1s spectra of N–doped graphene synthesized at 230–600 °C.

Temperature (^o^C)	Total N (at %)	Pyridinic N (eV)	Graphitic N (eV)	Pyrrolic N (eV)	Oxidation N (eV)	Normalization with graphitic N	Normalization with pyrrolic N
230	7.3	398.4	400.9			1:0.104	
300	8.5	398.3	400.9			1:0.261	
400	1.7		402.4	399.9			1:0.637
500	3.6		401.8	399.6	406.1		1:0.159
600	8.2		402.1	399.9	405.6		1:0.239
