# Supplementary material for: Tunable electronic properties of graphene through controlling bonding configurations of doped nitrogen atoms
Source: Sci Rep. 2016 Jun 21;6:28330. doi: 10.1038/srep28330 (PMC4914851; doi:10.1038/srep28330)
Supplement: Supplementary Information [file srep28330-s1.pdf]

Supporting information:

Tunable electronic properties of graphene through controlling bonding configurations of doped nitrogen atoms

Jia Zhang, [1§] Chao Zhao,[2§] Na Liu, [1] Huanxi Zhang, [1] Jingjing Liu, [1] Yong Qing Fu, [2,3] Bin Guo, [1] Zhenlong Wang,[1,4]* Shengbin Lei, [1] PingAn Hu[1,4]*

[1] Key Laboratory of Micro-systems and Micro-structure Manufacturing, Harbin Institute of Technology, Ministry of Education, No. 2 Yikuang Street, Harbin, 150080, China
[2] Faculty of Engineering & Environment, Northumbria University, Newcastle upon Tyne, NE1 8ST, UK

[3] Institute of Fundamental and Frontier Sciences, University of Electronic Science and Technology of China, No.4, North Jianshe Road, Chengdu, China

[4] State Key Laboratory of Robotics and System, Harbin Institute of Technology, No. 2 Yikuang Street, Harbin, 150080, China

[§]Jia Zhang and Chao Zhao have equal contribution to this work.

Corresponding Author:

Prof. Hu e–mail: hupa@hit.edu.cn

Prof. Wang e–mail: wangzl@hit.edu.cn

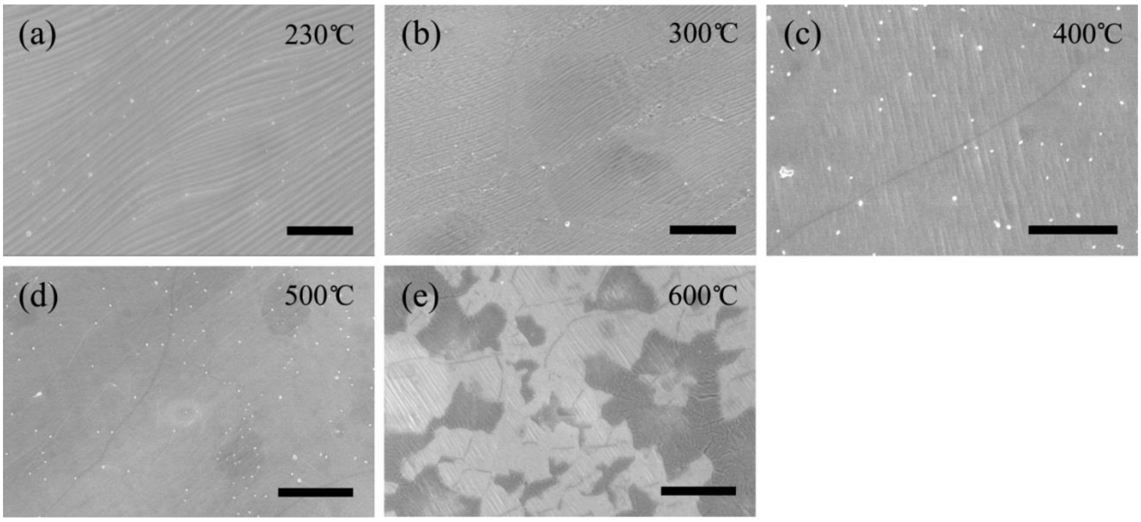

Figure S1. SEM image of N–doped graphene on Cu foils grown at temperatures in the range of 230–600 ^{o}C, scale bar=20 μm.

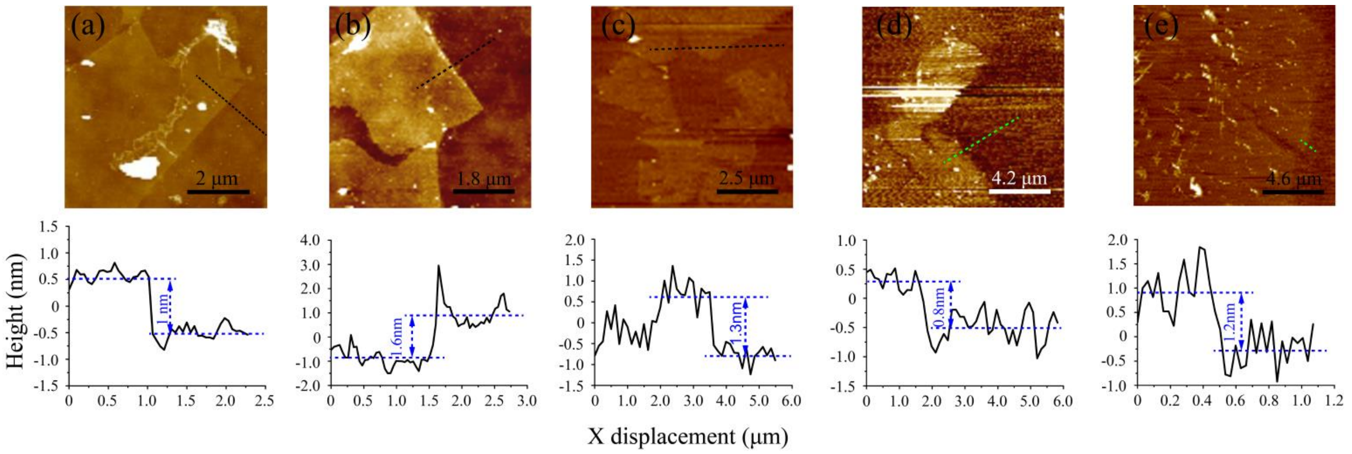

Figure S2. AFM images and cross-section profiles of the N–doped graphene on the SiO_2/Si substrate grown at temperatures of (a) 230 °C, (b) 300 °C, (c) 400 °C, (f) 500 °C and (e) 600 °C, respectively.

Table S1 Detailed parameters of Raman spectra collected from N–doped graphene grown at temperatures of 230–600 °C

Growth temperature (°C)	Position D band (cm^{-1})	Position G band (cm^{-1})	fwhm of G band (cm^{-1})	Position 2D band (cm^{-1})	Position D' band (cm^{-1})	fwhm of 2D band (cm^{-1})	I_{2D}/I_G	I_D/I_G	* L_a (nm)
230	1343.2	1588.1	29.2	2682.8	1626.6	33.1	2.57	1.21	8.91–11.86
300	1343.9	1589.0	24.3	2681.7	1625.9	33.9	2.46	1.14	9.56–12.71
400	1344.4	1590.2	20.3	2680.3	1626.0	29.2	2.50	0.48	14.73–19.59
500	1342.5	1590.0	27.0	2681.4	1626.3	36.2	2.25	1.66	7.92–10.53
600	1341.4	1585.6	27.2	2680.0	1625.0	36.6	2.26	1.91	7.38–9.82

L_D is defined as in–plane crystallite size, which is determined by an empirical formula reported by *A. C. Ferrari et al.* [1]

$$L_D^2(nm^2) = (1.8 \pm 0.5) \times 10^{-9} \lambda_L^4 \left(\frac{I_D}{I_G}\right)^{-1}$$

Where λ is the Raman excitation wavelength (532 nm in this work).

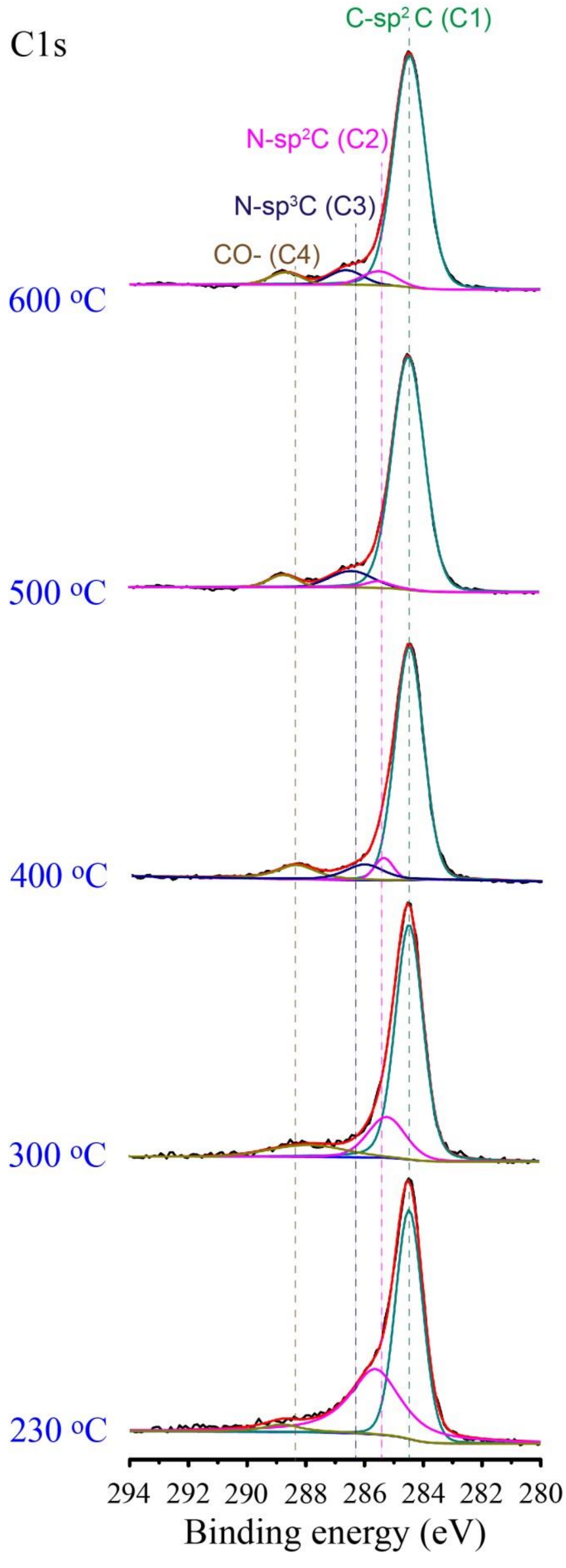

Figure S3 High–resolution XPS spectra of C1s of doped graphene grown at different temperatures (230~600 °C).

Table S2 Detailed parameters of C1s spectra of N–doped graphene synthesized at 230–600 ^{o}C.

Temperature (^{o}C)	C1	C2	C3	C4	C1(%)	C2(%)	C3(%)	C4(%)
230	284.5	285.2		288.0	73.4	17.3		9.3
300	284.5	285.6		288.8	55.4	40.9		3.7
400	284.5	285.4	286.2	288.4	77.3	5.6	8.7	8.4
500	284.6	285.4	286.3	288.4	84.9	2.6	8.6	3.9
600	284.5	285.5	286.5	288.7	85.6	5.1	5.5	3.8

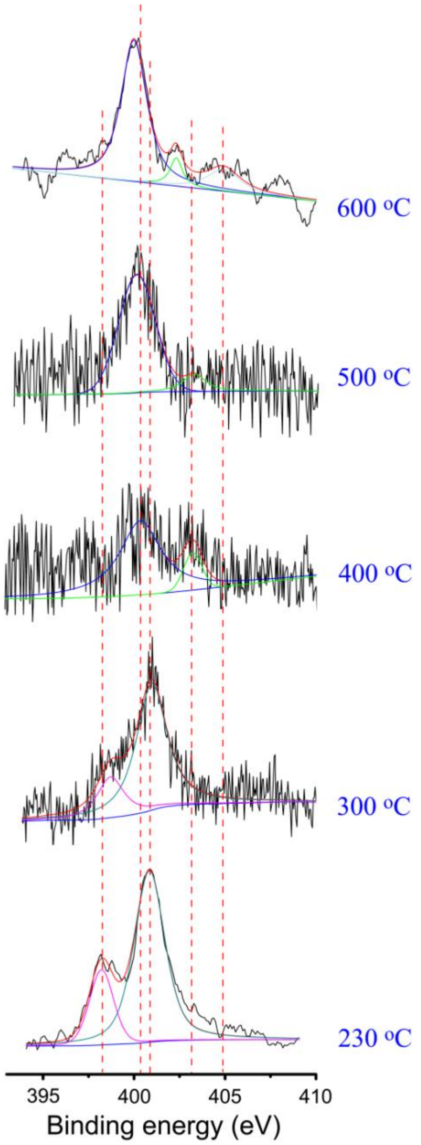

Figure S4 High–resolution XPS scan of N1s of doped graphene grown at different temperatures (230~600 ^{o}C).

Table S3 Detailed parameters of N1s spectra of N–doped graphene synthesized at 230–600 ^{o}C.

Temperature (^{o}C)	Total N (at%)	Pyridinic N (eV)	Graphitic N(eV)	Pyrrolic N (eV)	Oxidation N (eV)	Normalization with graphitic N	Normalization with Pyrrolic N
230	6.8	398.2	400.8			1:0.303	
300	3.6	398.7	401.0			1:0.286	
400	1.36		403.2	400.4			1:0.150
500	2.6		403.1	400.1			1:0.129
600	7.9		402.3	400.0	404.9		1:0.306

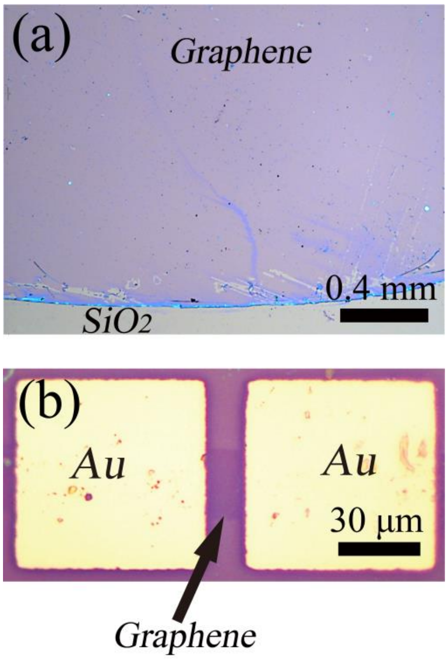

Figure S5 (a) A typical optical image of N–doped graphene film on 300 nm SiO_2/Si wafer. (b) Optical image of as–made FET device.

Reference

(1) Cancado, L. G.; Jorio, A.; Martins Ferreira, E. H.; Stavale, F.; Achete, C. A.; Capaz, R. B.; Moutinho, M. V. O.; Lombardo, A.; Kulmala, T. S.; Ferrari, A. C. Quantifying Defects in Graphene via Raman Spectroscopy at Different Excitation Energies. *Nano Lett*. **2011**, *11*, 3190–3196.
